# Pharmacokinetics of Esomeprazole in Critically Ill Patients

**DOI:** 10.3389/fmed.2021.621406

**Published:** 2022-02-07

**Authors:** Yanyan Xu, Xin Tian, Wei Wang, Weiqiang Tian, Tao Zhang, Jian Sun, Qingyun Zhou, Chuxiao Shao

**Affiliations:** ^1^Department of Pharmacy, Lishui Municipal Central Hospital, Lishui, China; ^2^Department of Intensive Care Medicine, Lishui Municipal Central Hospital, Lishui, China; ^3^Department of General Surgery, Lishui Municipal Central Hospital, Lishui, China

**Keywords:** esomeprazole, PK parameters, proton pump inhibitors, CYP2C19, ICU patients

## Abstract

**Background:**

Esomeprazole, a potent proton pump inhibitor (PPI), is widely used for the prevention of stress ulcers in intensive care unit (ICU) patients.

**Objective:**

This study investigates the pharmacokinetics (PK) of esomeprazole in critically ill patients.

**Methods:**

The study included eligible adult ICU patients who received endotracheal intubation assisted mechanical ventilation for more than 48 h and had at least an extra risk factor for stress ulcers. All enrolled patients received once-daily intravenous (IV) esomeprazole 40 mg. After the first dose of esomeprazole was administrated, serial blood samples were collected at 3, 5, 15, 30 min and 1, 2, 4, 6, 8, and 10 h. The total sample concentrations of esomeprazole were measured by UPLC-MS/MS. Esomeprazole PK parameters were analyzed using noncompartmental analysis.

**Results:**

A total of 30 patients were evaluable. Mean age and body mass index (BMI) were 61.97 years and 23.14. PK sampling on the first dose resulted in the following median (IQR) parameters: AUC_0−∞_ 8.06 (6.65–9.47) mg·h/L; MRT_0−∞_ 4.70 (3.89–5.51) h; t_1/2_ 3.29 (2.7–3.87) h; V 24.89 (22.09–27.69) L; CL 6.13 (5.01–7.26) L/h; and C_max_ 2.56 (2.30–2.82) mg/L.

**Conclusions:**

According to the label of esomeprazole, our study showed different esomeprazole PK parameters in ICU patients compared with healthy volunteers. Esomeprazole has unique pharmacokinetic parameters in critically ill patients.

## Introduction

Stress-induced ulcers are extremely common in ICU patients. Because these patients usually have risk factors for stress ulcers, such as coagulopathy, mechanical ventilation for at least 48 h, a Glasgow Coma score of ≤10, or multiple organ dysfunction syndrome (MODS) (1, 2). Approximately 75–100% of ICU patients experience mucosal injury within 24 h of ICU admission. Among them, 5–25% of ICU patients may have obvious bleeding if they do not receive drugs to prevent stress ulcers (3). Multiple studies have reported that stress-related ulcer bleeding may increase the mortality risk of ICU patients (4, 5). Therefore, stress ulcer prophylaxis (SUP) is recommended by many guidelines and expert consensuses, such as the Surviving Sepsis, Stress Ulcer Prophylaxis Campaign, and the Expert Suggestions of Prevention and Treatment of Stress Ulcer (2, 6).

PPIs and histamine 2 receptor antagonists (H2RA) are the two kinds of acid-suppressive agents most commonly used in SUP. However, Meta-analyses show that the PPIs used for SUP can significantly reduce the rate of ulcer bleeding compared with H2RA (7). Esomeprazole is the first (*S*)-isomer of the PPIs family approved for listing in the United States and major European countries in 2001 with oral and intravenous formulations (8). Esomeprazole is extensively metabolized in the liver by the cytochrome P450 (CYP) enzyme system. The major part of esomeprazole's metabolism is dependent upon the CYP2C19 isoenzyme, which forms the hydroxy and desmethyl metabolites. The remaining amount is dependent on CYP3A4 which forms the sulphone metabolite. In CYP2C19 extensive metabolizers, esomeprazole is inactivated at a faster rate. In contrast, CYP2C19 poor metabolizers have approximately twice the level of exposure to esomeprazole. Although genetic variation in CYP2C19 influences the plasma concentration of esomeprazole, the Dutch Pharmacogenetics Working Group of the Royal Dutch Association for the Advancement of Pharmacy (KNMP) recommends that esomeprazole dosage is not changed when CYP2C19 is normal, intermediate, or poorly metabolized (9). Patients with severely impaired hepatic function had a lower rate of metabolism. The AUC was 76% higher than in patients with Gastro-Oesophageal Reflux Disease (GORD) (10).

Numerous clinical trials have demonstrated that esomeprazole has a more pronounced acid-suppressive effect and fewer adverse events than other PPIs (11–13). According to several comparative studies of the acid suppressant efficacy of PPIs, the pharmacokinetic parameters of PPIs are positively correlated with their acid-suppressive effect. Among all PPIs products on the market, esomeprazole has superior pharmacokinetic characteristics and had been proven to be the best acid control effect in the clinical application (10). One study shows that the AUC of esomeprazole was almost 2-fold higher than that of omeprazole at the same dosage (14). The result showed that esomeprazole has a better acid-suppressive effect with a longer duration of intragastric pH > 4 (14). Previous studies on the pharmacokinetics of esomeprazole were carried out in healthy, elderly, patients with symptomatic GORD and patients with hepatic impairment. However, fewer PK parameters of esomeprazole in ICU patients were reported. ICU patients were critically ill with multiple-organ dysfunction, hypoalbuminaemia, and extracorporeal clearance techniques. Drug pharmacokinetic characteristics are often different from healthy subjects. Therefore, the objective of this trial was to describe the PK of a single dose of i.v. esomeprazole in critically ill patients.

## Materials and Methods

### Study Design

This open-label, single-treatment exploratory trial of IV esomeprazole was conducted in an Intensive Care Unit with critically ill patients with at least one additional risk factor for stress ulcer (Clinical Trial Registry, *ChiCTR1800018516*). The study protocol was approved by the Ethics Committee of Lishui Hospital of Zhejiang University (*Ethical Review of Clinical Research-2016-43*) and adhered to the tenets of the Declaration of Helsinki. Informed consent was obtained from the patients or the patients' legally authorized representative.

Patients aged 18–89 years who were admitted to ICU and receiving esomeprazole for the prophylaxis of stress-related mucosal disease were eligible to participate. Exclusion criteria included lactation or pregnancy, clinical diagnosis of treat peptic ulcer bleeding, peptic/stomach ulcer, gastroesophageal reflux disease, and Zollinger-Ellison syndrome, or the dosage of esomeprazole >40 mg in 1 day for other purposes. Patients also were excluded if they had a history of treated peptic ulcer bleeding, peptic/stomach ulcer, gastroesophageal reflux disease, and Zollinger-Ellison syndrome. All eligible patients received IV esomeprazole 40 mg once a day by the site nurse from the central vein. Each dose was injected slowly with 5 min. After IV esomeprazole 40 mg was administered, blood samples of 1.0 mL were drawn from the basilic vein: at 3, 5, 15, 30 min and 1, 2, 4, 6, 8, and 10 h. All blood samples were stored at room temperature for 30 min after collection and then centrifuged at 1500 *g* for 10 min at room temperature. Plasma was aspirated and transferred into a labeled 1.5 mL Eppendorf tube and stored at −80°C immediately after aspiration until drug assay. Patients were eligible if the PK curve was completed.

### Baseline Parameters

Upon inclusion, the baseline parameters of each patient were registered: gender, age, weight, BMI, alanine transaminase (ALT), aspartate transaminase (AST), blood urine nitrogen (BUN), creatinine clearance rate (CCR), APACHE II score (within 24 h of ICU admission), and Child-Pugh class.

### Sample Measurement

Blood samples were collected according to the scheduled time and the concentration of esomeprazole in plasma and were determined by UPLC-MS/MS according to a previous method [15]. The chromatographic column was the ACQUITY UPLC BEH C18 column (2.1 × 50 mm, 1.7 um). Esomeprazole was separated by gradient elution, which consisted of mobile phase A acetonitrile and A 0.1% formic acid and 5 mM ammonium formate in water. Gradient condition was detailed as follows: total run time was 3 min. Initially, mobile phase A was sustained as 20% from 0 to 0.7 min. Ten, A was reached to 80% for the 0.9 min. Ten 80% of mobile phase A was maintained for 0.5 min. Next, the mobile phase A was drawn back to 20% for 0.7 min and equilibrated as 20% for the 2 min. The flow rate was 0.40 ml/min, and the column was 40°C. Detection was conducted with a triple quadrupole tandem mass spectrometer equipped with positive electrospray ionization (ESI) by multiple reactions monitoring (MRM) of the transitions. The ion transitions were m/z 346.2 >198.0 for esomeprazole and m/z 285.1 > 193.1 for diazepam (internal standard).

### CYP2C19 Genetic Analysis

Genetic polymorphism of cytochrome CYP2C19 was detected by DNA microarray, which was reported in our previous study (15). In brief, 4 mL of whole blood samples were collected from each patient; the DNA was extracted from the blood using a Blood Genomic DNA Extraction Kit, then the concentration and purity of extracted DNA was determined by spectrophotometry. The variants of the CYP2C19 gene were detected by a commercially available kit (BaiO Technology Co, Ltd., Shanghai, China). Six genotypes of CYP2C19 were classified as three metabolic phenotypes. CYP2C19 genotype of ^*^1/^*^1 is a normal metabolizer (NM). The intermediate metabolizer (IM) includes the CYP2C19 genotype of ^*^1/^*^2 and ^*^1/^*^3. The poor metabolizer (PM) includes CYP2C19 ^*^2 /^*^2, ^*^2/^*^3 or ^*^3/^*^3.

### Pharmacokinetic Analyzes

PK parameters of esomeprazole were calculated according to the plasma concentration-time profiles, which were analyzed by a noncompartmental model analysis in DAS 3.2.8 (Drug and Statistics 3.2.8, Shanghai China). Pharmacokinetic analyses were performed with evaluable data from patients who were eligible for the study and had a sufficient number of data points. The areas under the concentration of esomeprazole in the plasma vs. the time curve from time zero to infinity (AUC _0−∞_) and the area under the respective first moment-time curve from time zero to infinity (AUMC_0−∞_) were calculated by the linear trapezoidal rule and the standard area extrapolation method. MRT was calculated as AUMC_0−∞_/AUC_0−∞_. The plasma clearance (CL) was estimated as Dose/AUC_0−∞_. Plasma terminal half-life (t_1/2_) was calculated as ln2/λz, while λz is the terminal slope of the log plasma esomeprazole concentration-time profile. V was calculated as MRT × CL. The maximum esomeprazole concentration (C_max_) was directly determined from the plasma esomeprazole concentration-time curves.

### Statistical Analysis

The values for pharmacokinetic variables were stated as estimates with 95% confidence intervals for the true geometric means. All continuous variables were tested for normality by the Kolmogorov-Smirnov test. The data of skewed distribution were transformed into the log-normal distribution. After normal testing, statistical analysis was conducted by SPSS 20.0 using ANOVA. The changes of six main pharmacokinetic parameters of both liver function classification and CYP 2C19 polymorphism were analyzed by multivariate analysis of variance. *P* < 0.05 was considered to be significantly significant.

## Results

### Patients

All 30 participants completed the study. Baseline characteristics are summarized in Table 1. The patients were two/thirds of men, aged 18–88 years, and had a mean APACHE II score of 21.10. Other baseline characteristics were: mean body mass index 23.14 kg/m^2^, mean Child-Pugh score 6.37, mean creatinine clearance rate 91.32 mL/min, and mean albumin 29.90 g/L. 63.33% of participants had three or more additional stress ulcer risk factors.

**Table 1:** Baseline characteristics of all participants.

	**Participants (*n* = 30)**
**Demographics**	
Gender, n(%)	
Male	20 (66.67)
Female	10 (33.33)
Age(years), Mean(range)	61.97 (18–88)
Elderly (≥65 years), n(%)	15 (50.00)
Weight(kg), Mean(range)	63.10 (42.50–80.00)
BMI(kg/m^2^), Mean(range)	23.14 (18.16–26.67)
**Clinical characteristics**	
APACHE II, Mean(range)	21.10 (11–30)
Child-Pugh score, Mean(range)	6.36 (5–9)
CCR(mL/min), Mean(range)	91.32 (10.67–207.82)
Albumin(g/L), Mean(range)	29.90 (16.50–38.50)
**Additional stress ulcer risk factor, n (%)**	
Respiratory failure	22 (73.33)
Coagulation dysfunction	2 (6.67)
Severe craniocerebral trauma	12 (40.00)
Multiple trauma	11 (36.67)
Post-major surgical procedure	19 (63.33)
Sepsis	10 (33.33)
Shock or persistent hypotension	5 (16.67)
Acute renal or liver failure	2 (6.67)
Multiple organ dysfunction syndrome (MODS) and/or multiple organ failure (MOF)	6 (20.00)

*BMI, body mass index; APACHE II, acute physiologic and chronic health evaluation II; CCR, creatinine clearance rate*.

### Esomeprazole PK

**Figure 1:**
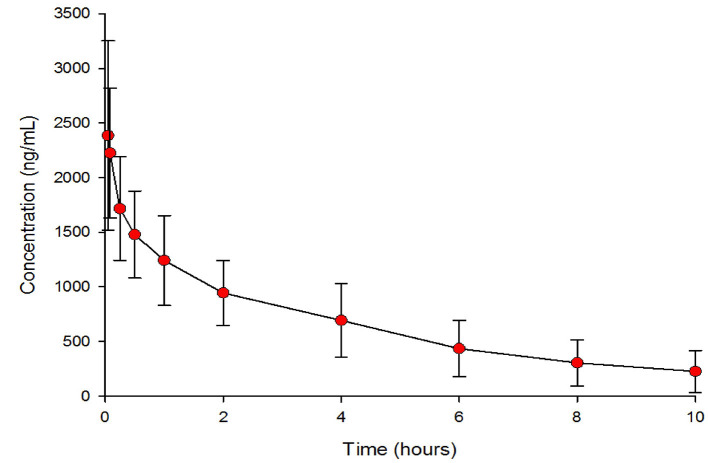
Esomeprazole plasma concentration after intravenous administrations of 40 mg dose age.

The geometric means (95% CI) of the PK parameters are presented in Table 2. Figure 1 shows the mean esomeprazole concentration profiles of a single i.v. dose of 40 mg over 10 h after administration.

**Table 2:** The pharmacokinetic parameters of esomeprazole.

	**Variable**	**Mean**	**95% CIs**
AUC_0−∞_	The area under the plasma concentration-time curve, mg·h/L	8.06	6.65–9.47
MRT_0−∞_	Mean retention time of the drug in the organism, h	4.70	3.89–5.51
t_1/2_	Half-life, h	3.29	2.71–3.87
V	Volume of drug distribution, L	24.89	22.09–27.69
CL	Clearance, L/h	6.13	5.01–7.26
C_max_	Maximum plasma drug concentration, mg/L	2.56	2.30–2.82

### Effect of CYP2C19 Metabolic Phenotype

The pharmacokinetic results were analyzed according to the CYP2C19 metabolic phenotype in Table 3. The PK parameters except for C_max_ were slightly different between NM, IM, and PM. NM and IM individuals had higher values of AUC_0−∞_, MRT_0−∞_, t_1/2_, and V. However, PM individuals had higher CL values than NM and IM individuals.

**Table 3:** Comparison of esomeprazole PK parameters with various CYP2C19 metabolic phenotype.

**Variable**	**Normal metabolizers (NM, *n* = 12)**	**Intermediate metabolizers (IM, *n* = 15)**	**Poor metabolizers (PM, *n* = 3)**	***P*-value**
AUC_0−∞_	8.29 (6.07–10.50)	8.34 (5.97–10.71)	5.76 (1.09–10.42)	0.56
MRT_0−∞_	5.08 (3.70–6.46)	4.58 (3.32–5.83)	3.84 (-1.27–8.96)	0.66
t_1/2_	3.67 (2.61–4.73)	3.18 (2.31–4.05)	2.33 (0.45–4.21)	0.40
V	26.30 (19.88–32.71)	24.00 (20.87–27.13)	23.73 (14.17–33.29)	0.72
CL	5.57 (4.07–7.08)	6.29 (4.33–8.25)	7.59 (0.23–14.95)	0.58
C_max_	2.56 (2.07–3.06)	2.53 (2.24–2.83)	2.67 (-0.45–5.78)	0.96

### Effect of Child-Pugh Grade

The Pharmacokinetic results were analyzed according to Child-Pugh grade in Table 4. According to Child-Pugh scoring criteria, 18 participants were in class A and 12 participants were in class B. The PK parameters of AUC_0−∞_, MRT_0−∞_, t_1/2_, and V were slightly different between Child A and Child B groups. Compared with the Child B group, values of AUC_0−∞_, MRT_0−∞_, t_1/2_, V, and C_max_ in the Child A group were low, but the CL value was high.

**Table 4:** Comparison of esomeprazole pharmacokinetic parameters with various Child-Pugh grade.

	**Variable**	**Child A (*n* = 18)**	**Child B (*n* = 12)**	***P*-value**
AUC_0−∞_	The area under the plasma concentration-time curve, mg·h/L	7.74 (6.07–9.40)	8.55 (5.70–11.39)	0.58
MRT_0−∞_	Mean retention time of the drug in the organism, h	4.25 (3.41–5.10)	5.38 (3.69–7.07)	0.17
t_1/2_	Half-life, h	2.91 (2.31–3.50)	3.86 (2.65–5.08)	0.10
V	Volume of drug distribution, L	23.51 (20.16–26.85)	26.97 (21.61–32.32)	0.22
CL	Clearance, L/h	6.29 (4.70–7.87)	5.90 (4.08–7.73)	0.74
C_max_	Maximum plasma drug concentration, mg/L	2.72 (2.34–3.10)	2.32 (2.00–2.65)	0.13

### Effect of Both Liver Function Classification and CYP 2C19 Polymorphism

The Pharmacokinetic results were analyzed with both Child-Pugh grade and CYP2C19 metabolic phenotype in Table 5. Table 6 showed that the variables of AUC_0−∞_, MRT_0−∞_, t_1/2_, V, CL, and C_max_ in Child-Pugh grade, CYP2C19 metabolic phenotype, and their interaction had no significant difference (*P* > 0.05).

**Table 5:** Comparison of esomeprazole pharmacokinetic parameters with various Child-Pugh grade and CYP2C19 metabolic phenotype.

**PK**		**Child A**	**Child B**
AUC_0−∞_	Normal metabolizers	8.62 (8.49–9.20)	7.31 (4.70–7.47)
	Intermediate metabolizers	6.97 (4.71–10.62)	10.81 (4.89–13.25)
	Poor metabolizers	6.40 (3.64–7.23)	/
MRT_0−∞_	Normal metabolizers	4.82 (3.75–6.61)	4.84 (3.27–7.32)
	Intermediate metabolizers	3.46 (2.73–5.55)	5.95 (2.93–8.67)
	Poor metabolizers	3.41 (2.03–6.08)	/
t_1/2_	Normal metabolizers	3.71 (2.35–4.82)	3.65 (2.17–5.94)
	Intermediate metabolizers	2.62 (1.63–3.63)	4.31 (2.20–5.83)
	Poor metabolizers	2.15 (1.68–3.16)	/
V	Normal metabolizers	24.82 (15.20–31.59)	28.82 (18.01–36.04)
	Intermediate metabolizers	24.57 (16.90–28.85)	23.50 (22.67–28.79)
	Poor metabolizers	25.21 (19.36–26.62)	/
CL	Normal metabolizers	4.64 (4.35–4.71)	5.47 (5.35–8.51)
	Intermediate metabolizers	5.76 (3.94–8.72)	3.70 (3.11–8.40)
	Poor metabolizers	6.25 (5.53–10.99)	/
C_max_	Normal metabolizers	2.84 (2.50–3.87)	2.20 (1.63–2.50)
	Intermediate metabolizers	2.45 (2.14–2.90)	2.71 (2.13–2.85)
	Poor metabolizers	2.08 (1.82–4.10)	/

**Table 6:** Tests of between Child-Pugh grade and CYP2C19 metabolic phenotype Effects.

**Source**	**Type III sum of squares**	**df**	**Mean square**	***F*-value**	**Sig**.
Dependent variable: AUC_0−∞_					
Corrected model	28.746	4	7.187	0.464	0.761
Intercept	1325.392	1	1325.392	85.624	0.000
Child-Pugh grade	0.858	1	0.858	0.055	0.816
CYP2C19 metabolic phenotype	16.855	2	8.427	0.544	0.587
Child-Pugh grade* CYP2C19	9.754	1	9.754	0.630	0.435
Dependent variable: MRT_0−∞_					
Corrected model	15.920	4	3.980	0.826	0.521
Intercept	493.836	1	493.836	102.459	0.000
Child-Pugh grade	5.203	1	5.203	1.080	0.309
CYP2C19 metabolic phenotype	1.302	2	0.651	0.135	0.874
Child-Pugh grade* CYP2C19	5.768	1	5.768	1.197	0.284
Dependent variable: t_1/2_					
Corrected model	10.768	4	2.692	1.117	0.371
Intercept	234.154	1	234.154	97.125	0.000
Child-Pugh grade	3.271	1	3.271	1.357	0.255
CYP2C19 metabolic phenotype	2.006	2	1.003	0.416	0.664
Child-Pugh grade* CYP2C19	2.397	1	2.397	0.994	0.328
Dependent variable: V					
Corrected model	110.896	4	27.724	0.454	0.768
Intercept	13605.724	1	13605.724	222.913	0.000
Child-Pugh grade	64.075	1	64.075	1.050	0.315
CYP2C19 metabolic phenotype	16.530	2	8.265	0.135	0.874
Child-Pugh grade* CYP2C19	10.168	1	10.168	0.167	0.687
Dependent variable: CL					
Corrected model	26.159	4	6.540	0.686	0.608
Intercept	842.319	1	842.319	88.402	0.000
Child-Pugh grade	0.167	1	0.167	0.018	0.896
CYP2C19 metabolic phenotype	10.757	2	5.378	0.564	0.576
Child-Pugh grade* CYP2C19	15.641	1	15.641	1.642	0.212
Dependent variable: C_max_					
Corrected model	2.654	4	0.663	1.478	0.239
Intercept	143.061	1	143.061	318.650	0.000
Child-Pugh grade	1.382	1	1.382	3.078	0.092
CYP2C19 metabolic phenotype	0.120	2	0.060	0.134	0.875
Child-Pugh grade* CYP2C19	1.402	1	1.402	3.122	0.089

## Discussion

According to the instructions of esomeprazole, the results of this study indicated that the esomeprazole PK parameters in critically ill patients were different from those in healthy volunteers. We observed highly variable PK parameters, in particular for the observed volume of drug distribution (V) and clearance (CL). Compared with the values reported for Chinese healthy volunteers, the value of V 24.89 (22.09–27.69) L in critically ill patients was 1.88 times higher and the value of CL 6.13(5.01–7.26) L/h was 35.85% lower (16).

Compared with healthy volunteers, many factors such as increased total body water and interstitial or ‘third space' fluid volumes, decreased albumin concentration, plasma pH changed, peripheral tissue penetration changed during septic shock, and increased permeability of the blood-brain barrier (BBB) that may influence drug distribution among critically ill patients (17). Therefore, V for hydrophilic drugs such as β-lactam antibiotics was increased in critically ill patients (18). The value of V (24.89 L) was nearly double that of healthy volunteers (13.32 L) (16), suggesting that esomeprazole was distributed more extensively in critically ill patients. Esomeprazole is a high protein binding drug and its protein binding rate is 97%. Therefore, unbound esomeprazole plasma concentration is increased in hypoproteinemia patients. According to the baseline characteristics of all participants, the mean albumin concentration was 29.90 g/L lower than normal values. Hypoproteinemia is one reason for high V. There were 10 participants of sepsis. High peripheral tissue penetration during septic shock also is one reason for high V. In addition, critically ill patients often have increased total body water and interstitial or “third space” fluid volumes were the most common factors that led to elevated V.

The CL in this study was 6.13 L/h, which was far lower than Chinese healthy volunteers 17.1 L/h (16). This means that esomeprazole was slowly cleared with longer residence time *in vivo*. Esomeprazole was the drug mainly metabolized by the liver CYP2C19 isoenzyme and nearly 80% was excreted from urine in the form of metabolites according to its drug instructions. CL could be affected by liver dysfunction, renal dysfunction, continuous renal replacement therapy (CRRT), and extracorporeal membrane oxygenation (ECMO) (17). There were 12 participants of Child-Pugh grade B, individuals with mild to moderate hepatic dysfunction could affect the reduced CL. CCR was 10.67–207.82 mL/min with high individual variability. Moderate-to-severe renal impairment also caused the reduced CL. In addition, the elevated V of esomeprazole was widely distributed in critically ill patients, which could lead to low clearance.

As t_1/2_ is proportional to V and inversely proportional to CL, the impact of a change in V during critical illness on the overall pharmacokinetics of esomeprazole likely depends on whether CL is affected. The mean t_1/2_ of esomeprazole in this study (3.29 h) was much higher than that reported in healthy volunteers (mean, 0.85 h) (10). This observation was consistent with the fact that clearance was reduced while the volume of distribution was increased, which would result in an increased elimination half-life (elimination rate constant *k* = CL/V) (15). This indicated that the retention time of esomeprazole in critically ill patients is prolonged.

AUC_0−∞_ is another important PK parameter in these special patients. The mean AUC_0−∞_ in this study (8.06 mg·h/L) was nearly more than three times the value described in healthy volunteers after a single intravenous dose of 40 mg (6.84 μmol·h/L) (16), when they were converted into unified units. Similarly, C_max_ was increased in critically ill patients. C_max_ of healthy volunteers (5.53 μmol/L) was only 74.6% of the value in critically ill patients (18). This was in line with the elevated V and reduced CL we observed. According to a previous report (10), esomeprazole had a clear AUC-effect relationship. AUC was correlated to the inhibitory effect on stimulated gastric acid secretion. Therefore, we speculated that the increased esomeprazole concentration exposure in blood with high AUC and C_max_ in critically ill patients could have a more effective acid control effect. The special pathological characteristics of critically ill patients caused AUC and C_max_ to change. Furthermore, the increased unbound fraction resulting from the low plasma albumin levels means that more free-drug was available for elimination, which is consistent with the increased C_max_ we observed in this study.

Esomeprazole is extensively metabolized in the liver by the human cytochrome P450 (CYP) enzyme system. According to the label for esomeprazole (Nexium IV), in patients with liver impairment, the dosage of esomeprazole should be adjusted based on Child-Pugh grade when esomeprazole was used to rebleeding of gastric or duodenal ulcers following therapeutic endoscopy. In order to evaluate the effect of the liver impairment on esomeprazole PK, we divided the participants into Child A group or Child B group according to their Child-Pugh score. Compared with the Child A group, AUC_0−∞_, MRT_0−∞_, t_1/2_, and V of esomeprazole in the Child B group were increased by 1.10, 1.27, 1.32, and 1.15-fold, but CL and C_max_ in Child B group were decreased to 94.80% and 85.29%. These PK parameters showed critically ill patients with severe liver impairment had wider distribution and longer retention of esomeprazole in the body. However, for the PK parameters, there was no significant difference between the two groups. This was because many factors could affect the metabolism of esomeprazole during critical illness, such as CYP2C19 genotype difference, administration of a CYP2C19 inhibitor or two drugs metabolized by the same CYP2C19 resulting in competitive inhibition, administration of an agent that induces CYP2C19, reduced hepatic or splanchnic blood flow as a result of shock, acute renal failure or increased protein binding to albumin or α_1_-acid glycoprotein, Gram-negative sepsis associated with the production of LPS and increased global or locoregional production of proinflammatory cytokines, surgical interventions and so on (17). In this study, the combined drugs of the enrolled cases were vasoactive drugs (norepinephrine), opioid analgesics (morphine, fentanyl), sedatives (midazolam, dexmedetomidine), antibiotics (imipenem cilastatin, piperacillin tazobactam, cefoperazone sulbactam, vancomycin), albumin and crystalloid fluid. These combined drugs with no clear reports of drug interaction with EPZ.

According to the label for esomeprazole (Nexium IV), CYP2C19 is the major metabolic enzyme, while the CYP2C19 poor metabolizers (PM) genotype is common in Asian populations with a ratio of 15–20%. The difference of the individual CYP2C19 genotype resulted in different PK and PD. At a steady state, the ratio of AUC in Poor Metabolizers to the rest of the population (Extensive metabolizers) is approximately 2 (14). In the current study, six genotypes of CYP2C19 were classified into three metabolic phenotypes, normal metabolizer (NM), intermediate metabolizer (IM), and poor metabolizer (PM). Compared with the PM group, the mean AUC_0−∞_ of esomeprazole were 1.44 and 1.45-fold higher than NM and IM groups. However, the ratio of AUC_0−∞_ between different CYP2C19 phenotypes was much lower than that reported in healthy volunteers (19). t_1/2_ in NM and IM groups were longer than PM group, while CL in NM and IM groups were lower than the PM group. The results of t_1/2_ and CL were inconsistent with the previous study (20). We speculated that the main reason was the small sample size with only three patients in the PM group.

There are two limitations of this study. One limitation is the small sample size of the PM group. Given the adequate sample size of the NM, IM, and PM groups, we can obtain the pharmacokinetic characteristics of critical patients with different CYP phenotypes. The other limitation is that the pharmacodynamics of the anti-acid effect and effective maintenance time at target pH after intravenous administration of esomeprazole were not designed. Further PK/ PD model clinical studies of esomeprazole plasma concentration with effects on 24 h intragastric pH levels are necessary to establish the scientific dosage regimen of critically ill patients. We recommend that further studies based on the population PK and PD are essential, for more data are required to promote the rational use of esomeprazole in critically ill patients.

## Conclusions

The PK of a single dose of 40 mg i.v. esomeprazole in critically ill patients was different from the PK data reported by previous studies in healthy volunteers receiving the same i.v. dose of 40 mg.

## Data Availability Statement

The datasets presented in this study can be found in online repositories. The names of the repository/repositories and accession number(s) can be found below: Figshare (https://doi.org/10.6084/m9.figshare.13141406.v2 and https://doi.org/10.6084/m9.figshare.17019536).

## Ethics Statement

The studies involving human participants were reviewed and approved by Ethics Committee of Lishui Municipal Central Hospital. The patients/participants provided their written informed consent to participate in this study.

## Author Contributions

CS, YX, and XT participated in the design of this study and they both performed the statistical analysis. WT and QZ tested the blood concentration. YX and WT carried out pharmacokinetic analyzes. WW, TZ, and JS managed patients and collected data. YX drafted the manuscript. All authors read and approved the final manuscript.

## Funding

This material is based upon work funded by the Beijing Medical and Health Foundation of China under grant no. YWJKJJHKYJJ-212, Zhejiang Provincial Natural Science Foundation of China under grant no. LY18H300007, High Level Talented Person Cultivating Program of Lishui Science and Technology Bureau under grant no. 2018RC06, and Zhejiang TCM science and technology program under grant no. 2013ZB148.

## Conflict of Interest

The authors declare that the research was conducted in the absence of any commercial or financial relationships that could be construed as a potential conflict of interest.

## Publisher's Note

All claims expressed in this article are solely those of the authors and do not necessarily represent those of their affiliated organizations, or those of the publisher, the editors and the reviewers. Any product that may be evaluated in this article, or claim that may be made by its manufacturer, is not guaranteed or endorsed by the publisher.
